# Surface Landmarks to Provide a Safe Ulnar Nerve Block in the Wrist: Anatomical Study and Literature Review

**Published:** 2020-10-19

**Authors:** Swapnil D. Kachare, Luke T. Meredith, Milind D. Kachare, Bradley J. Vivace, Christina N. Kapsalis, Claude Muresan, Joshua H. Choo, Morton L. Kasdan, Bradon J. Wilhelmi

**Affiliations:** ^a^Division of Plastic and Reconstructive Surgery, Department of Surgery; ^b^School of Medicine, University of Louisville, Louisville, KY; ^c^Department of Surgery, Robert Wood Johnson Medical School, New Brunswick, NJ; ^d^Division of Plastic and Reconstructive Surgery, Department of Surgery, University of North Carolina, Chapel Hill; ^e^Christine M. Kleinert Institute for Hand and Microsurgery, Louisville, KY

**Keywords:** ulnar nerve block, wrist block, local anesthesia, safe injection, iatrogenic injury

## Abstract

**Introduction:** Use of local anesthesia in awake patients undergoing hand surgery has become increasingly popular. A thorough understanding of local anatomy, such as the distal wrist for ulnar nerve block, is required to provide safe blockade. We sought to conduct an anatomic study of the distal wrist and review cadaveric studies describing various techniques for ulnar nerve block. **Methods:** Dissection of fresh-frozen cadaver forearms at the University of Louisville Robert Acland Fresh Tissue Lab assessing relationships between the flexor carpi ulnaris tendon and the ulnar nerve and the ulnar artery was performed. Three cadaveric studies on ulnar nerve blockade using the ulnar, volar, and/or transtendinous technique were identified and reviewed. **Results:** A total of 16 cadaver forearms of equal male to female ratio were obtained. The ulnar nerve was noted to be directly posterior to the flexor carpi ulnaris tendon in 15 (93.8%) forearms, with 1 (6.3%) specimen having the nerve extend along the ulnar border of the flexor carpi ulnaris. The ulnar artery was radial to the ulnar nerve 1 cm proximal to the pisiform in all specimens. In all 3 cadaveric studies, only the ulnar technique was associated with no ulnar artery and/or ulnar nerve injury. **Conclusion:** Knowledge of distal wrist anatomy can help minimize risk of iatrogenic injury during local blockade. On review, the ulnar approach provides the safest method for ulnar nerve block.

## SUMMARY:

Knowledge of distal wrist anatomy is required to execute a safe ulnar nerve block. We performed a cadaveric anatomical study as well as a literature review of cadaveric studies describing various techniques for ulnar nerve blockade at the wrist. The data suggest that an ulnar approach provides the safest method.

Anesthesia modalities in hand surgery range from local blockade to general anesthesia. Recently, wide awake local anesthesia no tourniquet (WALANT) hand surgery has gained popularity, utilizing local anesthetics combined with epinephrine for hemostasis.^1^^-^4 This method provides safe and effective anesthesia while decreasing costs and operating room time.^5^^-^7 However, knowledge of local anatomy to ensure precision of injection is required to avoid iatrogenic injury.^8^

When anesthetizing the ulnar nerve (UN) as part of a wrist block, injury and/or intra-arterial injection of the ulnar artery (UA) can occur due to their proximity in the distal forearm.^9^ The UN commonly courses radial to the UA on the surface of the flexor digitorum profundus and deep to the flexor carpi ulnaris (FCU) in the proximal two-thirds of the forearm. As the UN approaches the distal third, it crosses ulnar to the UA, giving off a dorsal cutaneous branch 3 to 5 cm proximal to the ulnar head and continues between the pisiform and the hook of hamate.^10^

Use of surface landmarks to predict nerve locations for injection in the upper limb had been described to avoid iatrogenic injury, with multiple anatomic and cadaveric studies focusing on the median nerve.^8^^,^^11^ However, limited cadaveric studies exist describing injection for UN blockade at the wrist, with no consensus on technique.^12^^-^14 We sought to review this literature as well as compare the findings with our data. Using the relationship of the UN to the UA, pisiform, and FCU, we aim to describe a safe location for injection of local anesthetic during UN blockade.

## METHODS

Fresh-frozen cadaveric upper extremities were obtained from the University of Louisville Robert Acland Fresh Tissue Lab. The UN was revealed by dissection, beginning at the cubital tunnel and progressing distally. The nerve was traced until it entered Guyon's canal. The relationships between the FCU, UN, and UA were recorded 1 cm proximal to the proximal aspect of the pisiform, corresponding to the wrist crease.

Upon literature review, 3 cadaveric studies for UN blockade were identified. In 2004, Lizamore et al^12^ inserted 2 needles into 57 cadaveric wrists, each with a volar approach and an ulnar approach. Upon dissection, the presence of UA puncture, relationship of the UN to the UA, and depth of needle (ie, distance of the medial FCU to the medial UN, via an ulnar approach) were measured.^12^ Then, in 2014, Varshney et al^13^ inserted a 1.5 inch 25 gauge needle proximal to the wrist crease in 40 cadavers, utilizing the FCU as a landmark, via a volar, transtendinous volar (TTV), or ulnar technique. They assessed the distance from the needle tip to the UN and UA and the number of arterial punctures.^13^ In addition, in 2014, Prithishkumar et al^14^ inserted two 18 gauge needles at a total depth of 7 mm, just proximal to the wrist crease in 12 cadavers, one via a volar approach and second via an ulnar approach. The following measurements were obtained: medial border of the FCU to the UA, medial border of the FCU to the UN, lateral border of the FCU to the UA, UN to UA, width of the FCU tendon, and tip of the needle to the UA via a volar approach and an ulnar approach.^14^

## RESULTS

### Cadaveric Study

**Figure 1:**
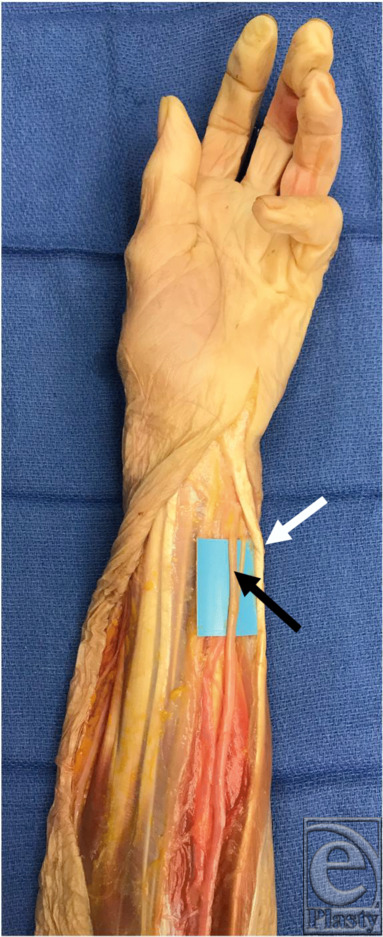
Ulnar nerve (black arrow) with the flexor carpi ulnaris (white arrow).

**Figure 2:**
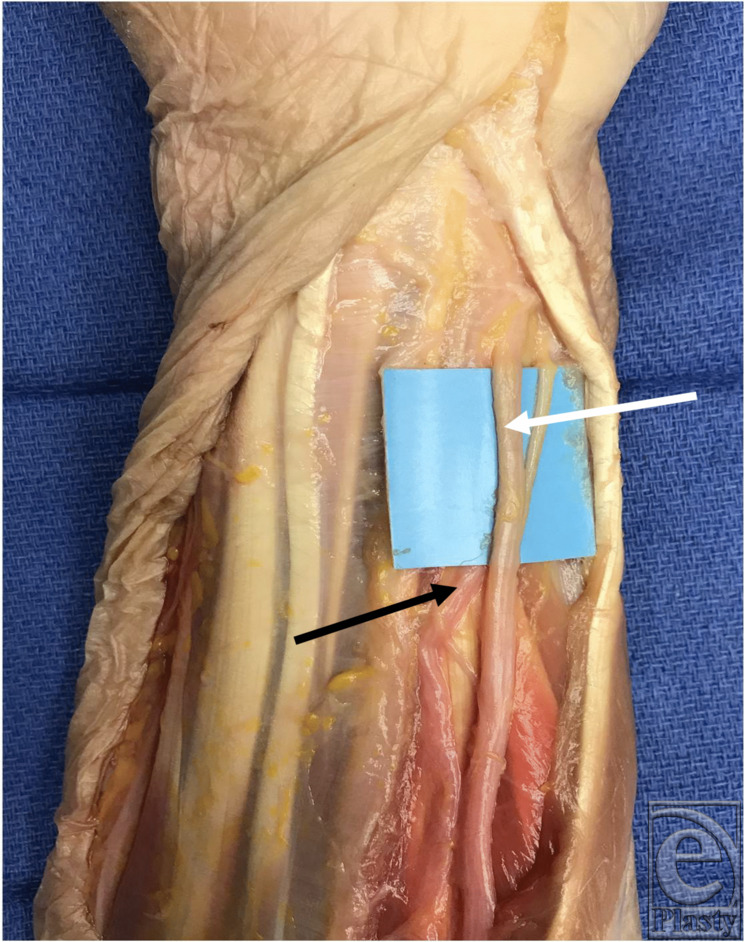
Ulnar artery (black arrow) radial to the ulnar nerve (white arrow) in the distal forearm.

A total of 16 (7 right and 9 left) cadaveric upper limbs were studied. Eight (50%) were female, and 15 (93.8%) were white (Table 1). The UN was noted to be directly posterior to the tendon of the FCU in 15 (93.8%) specimens (Fig 1). In one (6.3%) specimen, the nerve was noted to be along the ulnar border of the FCU (Table 1). The circumference of the distal wrist ranged from 12.5 to 19 cm, with a mean of 15.3 cm (Table 1). The UA was radial to the UN 1 cm proximal to the pisiform in all specimens (Fig 2).

### Literature Review

#### Alternative approaches for regional ulnar nerve blockade: A cadaveric study

Lizamore et al^12^ identified that the UN was medial to the UA in 53 (92.9%), medial and posterior in 3 (5.3%), and anterior in 1 (1.8%) of the wrists at the crease. UA injury was present in 21 (36.8%) of the wrists in a volar approach versus 0 in an ulnar approach. On an ulnar approach, the mean distance between the ulnar border of the FCU tendon and the ulnar border of the UN (depth of needle insertion) was 0.88 cm (range, 0.46-1.39 cm).^12^

#### A cadaveric study comparing the 3 approaches for ulnar nerve block at the wrist

In comparing volar, ulnar, and TTV techniques, Varshney et al^13^ identified a significant difference (*P* = .001) in mean distances between needle tip to the UN and needle tip to the UA. Needle tip to the UA was 0.92 mm for volar, 3.96 mm for TTV, and 7.14 mm for ulnar techniques, while needle tip to the UN was 0.71 mm for volar, 3.61 mm for TTV, and 6.31 mm for ulnar techniques. On a volar approach, the UA was punctured in 14 (35%) wrists, with no penetration via ulnar and TTV techniques. Neural puncture occurred in 16 (40%) in the volar approach and 2 (5%) in the TTV approach, with no injury via the ulnar approach.^13^

#### Comparison of the volar and medial approach in peripheral block of the ulnar nerve at the wrist: A cadaveric study

Prithishkumar et al^14^ demonstrated that the UN was always deep to the ulnar aspect of the FCU tendon; however, the UA, while always radial to the UN, varied in its relationship to the FCU. They describe 3 relationship types; type I (33.3%): UA deep to the FCU; type II (50%): UA just radial to the radial aspect of the FCU tendon; and type III (16.7%): UA far radial to the FCU radial border. UA puncture rate was 50% on the volar approach and 0% on the ulnar approach.^14^

## DISCUSSION

A thorough understanding of the relationships between the UN and surface landmarks at the wrist is important in order to safely perform an UN block. The use of ultrasound has been reported to assist in performing UN injections^15^; however, its effectiveness is dependent on ultrasound availability and user ability and should not replace a surgeon's knowledge of the anatomy. This study focuses on the location of the UN in relation to the UA, pisiform, and FCU in the distal forearm in order to guide performance of an ulnar wrist block.

Prior to discussion of a proper technique, it is important to note that although local nerve blocks are relatively safe when compared with general anesthesia, there are inherent associated risks. Damage to the nerve or adjacent artery during injection of local anesthetics can be particularly debilitating, and introduction of the local anesthetic into the bloodstream can have devastating effects.^16^ Many factors influence the risk and degree of local anesthetic systemic toxicity (LAST), including comorbidities, medication interactions, the type, amount, location, and route of delivery of the anesthetic, as well as the time until detected. Because of the increasing use and interest in local anesthesia, the American Society of Regional Anesthesia and Pain Medicine (ASRA) has continually published and updated a checklist for identifying and managing LAST, with the most recent being published in 2018.^17^^,^^18^

**Figure 3:**
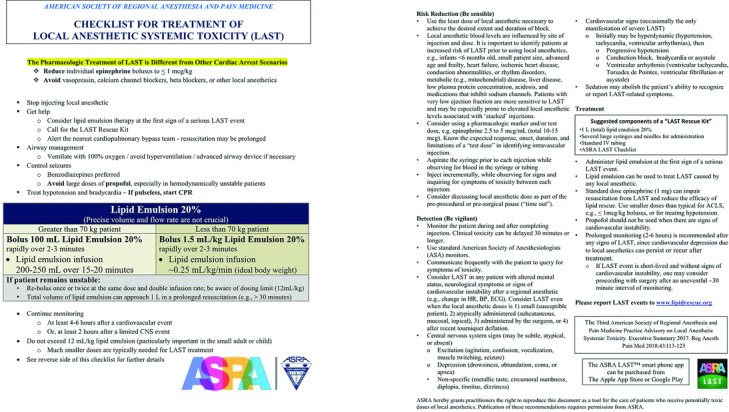
ASRA checklist for the treatment of LAST. LAST indicates local anesthetic systemic toxicity; CPR, cardiopulmonary resuscitation; CNS, central nervous system; HR, heart rate; BP, blood pressure; ECG, electrocardiogram. Used with permission from the American Society of Regional Anesthesia and Pain Medicine.

Classically, the description of LAST includes minor central nervous system symptoms that continually progress, with cardiac toxicity following. This progression, however, may be accelerated or bypassed during intravascular injection. Once recognizing this, it is vital to initiate treatment immediately. Of note, the ASRA stresses that although this may result in findings that trigger the standard ACLS protocols, the cause is in fact different and requires a modified approach. Additional to the recommended treatment algorithm, it is recommended to have a LAST Rescue Kit available and the ASRA LAST Checklist (Fig 3).^17^^,^^18^

Another well-known risk during local anesthetic injection is peripheral nerve injury.^19^ Factors affecting the extent of nerve injury include type and size of the needle, angle of insertion, pressure of injection, and dose of anesthetic injected. Local anesthetics have a direct toxic effect on nerves when injected in the intrafascicular plane. In a study by Farber et al,^20^ nerves injected with bupivacaine, lidocaine, or ropivacaine underwent neural fibrosis and loss of myelinated fibers, whereas injection of saline alone caused no severe injury. The effects of nerve injury during local anesthetic injection include loss of sensation or motor function, pain, and paresthesia.^20^^,^^21^ Using a small needle, and having the patient awake during injection, has been proposed as a method to avoid nerve injury. This allows for observation of the patient's reaction to the introduction of the needle where pain or paresthesia may indicate the needle is coming into direct contact with the nerve.^8^

To prevent such complications, it is essential to understand the anatomic relationships between the UA, UN, and FCU. Focusing on the relationship between the UA and the UN, Prithishkumar et al^14^ and Varshney et al^13^ demonstrated that the UA was radial to the nerve in all cadaveric specimens, 12 and 40, respectively,^13^^,^^14^ which was consistent with our findings in 16 cadaveric specimens. Lizamore et al^12^ demonstrated similar findings in 56 of 57 specimens, with one being posterior to the nerve.^12^ Although there was slight variation in the neurovascular relationship, the artery was never found to be on the ulnar side of the nerve.^12^^-^14 Regarding the relationship between the FCU and the UA, Prithishkumar et al^14^ described 3 types, with 33% of UAs being under the FCU (type 1), 50% just radial to the FCU (type II), and 16.7% further radial from the FCU (type III).^14^ While not distinguishing between types, Varshney et al^13^ demonstrated that for all the specimens on a volar approach, the mean distance from the needle tip was closer to the UN than to the UA, suggesting the UA was primarily in a type II or III position.^13^ Lizamore et al^12^ also specified a relationship between the UA and the FCU, but their findings also seemed to describe a type II or III position.^12^ We identified the UA to be located in all 3 types as described by Prithishkumar et al.^14^ Finally, comparing the relationship between the FCU and the UN at the wrist, there was also variance in findings. Varshney et al^13^ described the nerve to be located radial to the FCU, which was similar the findings of Lizamore et al.^12^ In contrast, Prithiskumar et al^14^ found the UN to be deep to the FCU tendon,^14^ similar to our findings, with one nerve (6.2%) located on the deep ulnar side of the FCU tendon.

In defining the techniques, the ulnar and volar approaches are the most classically used methods for UN blockade at the wrist. In the ulnar approach, the needle is inserted parallel to the proximal wrist crease, just dorsal to the FCU and perpendicular to the long axis of the arm. In the volar approach, the needle is inserted perpendicular to the long axis of the arm at the proximal wrist crease along the radial border of the FCU. An alternative approach is the TTV approach, which involves inserting the needle perpendicular to the long axis of the arm at the proximal wrist crease through the midportion of the FCU tendon until loss of resistance indicates the needle has traversed the tendon. In all 3 methods, aspiration prior to injection is important in order to avoid intra-arterial injection.^12^^-^14

Utilizing the volar approach, the rate of arterial puncture ranged from 35% to 50%, with 40% risk of UN injury as described by Varshney et al.^13^ In contrast, the ulnar approach was associated with a 0% risk of arterial puncture in all 3 studies and a 0% risk of nerve injury as long as the needle was inserted less than 10 mm. Given these findings, both Lizamore et al^12^ and Prithishkumar et al^14^ recommended the ulnar approach. Regarding the TTV approach, Varshney et al^13^ found a 0% risk of arterial puncture and a 5% risk of neural injury, which they stated were not significantly different from the risk of neural injury via the ulnar approach (*P* = .49). Because of a shorter distance between the needle tip and the UN via the TTV approach versus the ulnar approach, which they suggest would result in a more effective block, Varshney et al^13^ recommended the TTV approach. Incorporating the findings from these 3 studies, as well as our anatomic findings, we favor the ulnar approach, as it is associated with both a 0% risk of UA puncture and an avoidable UN injury with limited needle insertion.^12^^-^14 This may be most important in patients who are unable to provide feedback as compared with awake patients who can provide immediate response for adjustment of needle depth, which is a variable factor, further confirmed by the large range in wrist circumference seen within our study.

There are inherent limitations to this cadaveric study, which precludes the component of immediate feedback, palpable UA, and a limited number of specimens. In addition, only one study discussed the TTV approach,^13^ limiting our conclusions regarding this approach. However, by assessing the cadaveric studies focusing on an UN block at the wrist in combination with our study population, we are able to better define anatomic relationships and the impact of these relationships on the various techniques for UN block.

Although there are innate risks involved with using local anesthetics, these risks can be mitigated through a thorough understanding of the anatomical relationships, along with the utilization of surface landmarks and proper injection techniques, with our preference being the ulnar approach.

## Figures and Tables

**Table 1:** Demographics and distal wrist anatomy; flexor carpi ulnaris*

Specimen	Gender	Race	Laterality	Distal wrist circumference, cm	Location of the ulnar nerve 1 cm proximal to the pisiform (distal wrist crease)
1	Male	African American	Right	17.7	Ulnar border of the FCU
2	Female	White	Right	12.5	Directly posterior to the FCU
3	Female	White	Left	12.9	Directly posterior to the FCU
4	Male	White	Right	16.8	Directly posterior to the FCU
5	Male	White	Left	18.2	Directly posterior to the FCU
6	Female	White	Left	13.6	Directly posterior to the FCU
7	Male	White	Left	19	Directly posterior to the FCU
8	Male	White	Left	17.2	Directly posterior to the FCU
9	Female	White	Left	15	Directly posterior to the FCU
10	Male	White	Right	15	Directly posterior to the FCU
11	Male	White	Right	16.5	Directly posterior to the FCU
12	Female	White	Left	13.2	Directly posterior to the FCU
13	Male	White	Right	16.5	Directly posterior to the FCU
14	Female	White	Left	14.0	Directly posterior to the FCU
15	Female	White	Left	13.0	Directly posterior to the FCU
16	Female	White	Right	14.0	Directly posterior to the FCU

*FCU indicates flexor carpi ulnaris.
